# Supplementary material for: A single-cell transcriptomic landscape of innate and adaptive intratumoral immunity in triple negative breast cancer during chemo- and immunotherapies
Source: Cell Death Discov. 2022 Mar 8;8:106. doi: 10.1038/s41420-022-00893-x (PMC8904804; doi:10.1038/s41420-022-00893-x)
Supplement: Supplementary file 1 — Supplementary Materials [file 41420_2022_893_MOESM1_ESM.docx]

**Supplementary Text**

**Automatic Cell Assignation Results**

Clusters 0, 13, 15, 18 and 19, in the 4T1 cell line, had the majority of cells automatically assigned (see materials and methods) to B cell population; clusters 1, 2, 3 ,4, 6, 7, 8 and 11 contained a high percentage of cells belonging to T cells; cells in clusters 9 and 10 were assigned to macrophages; finally, cluster 5 contained a high percentage of neutrophils, while cluster 14 presented a high percentage of NK cells. Low percentages of unassigned cells were observed in clusters 12, 15 and 19, and very few cells (17 cells on 22,403 cells for 4T1 and 21 on 26,245 cells for EMT6) in some clusters were automatically assigned to cell populations that were Cd45^-^ cells such as fibroblast and epithelial cells ([Fig. S3](https://docs.google.com/document/d/1-rJ-QR2CKkKYkGkVqR04yscehPeO4lWL/edit#bookmark=id.gjdgxs)A). Likely, it was the result of either an ambiguous assignment due to the lack of real label for these cells^1^ or a technical barrier related to the purity at sorting level (see Materials and Methods). These together with a need of validation were the reasons for the manual exploration (see materials and methods).

EMT6 automatic cell assignment ([Fig. S3](https://docs.google.com/document/d/1-rJ-QR2CKkKYkGkVqR04yscehPeO4lWL/edit#bookmark=id.gjdgxs)B) identified clusters 0, 4, 7, 8, 9, 12 and 13 as macrophages; the 3, 10, 14 and 18 clusters were mainly composed of T cell populations; while cells in clusters 2 and 5 were assigned to monocytes. Finally, clusters 1, 15 and 16 were composed mainly by B cells. Compared to 4T1, a higher percentage of unassigned cells was present in EMT6; specifically in clusters 9, 13, 18 and 21. Likely, this was associated with a low expression of gene signature markers revealed by *SingleR*^1^.

**Bibliography**

1. Aran, D. *et al.* Reference-based analysis of lung single-cell sequencing reveals a transitional profibrotic macrophage. *Nature Immunology* **20**, 163–172 (2019).

**Supplementary Figures**


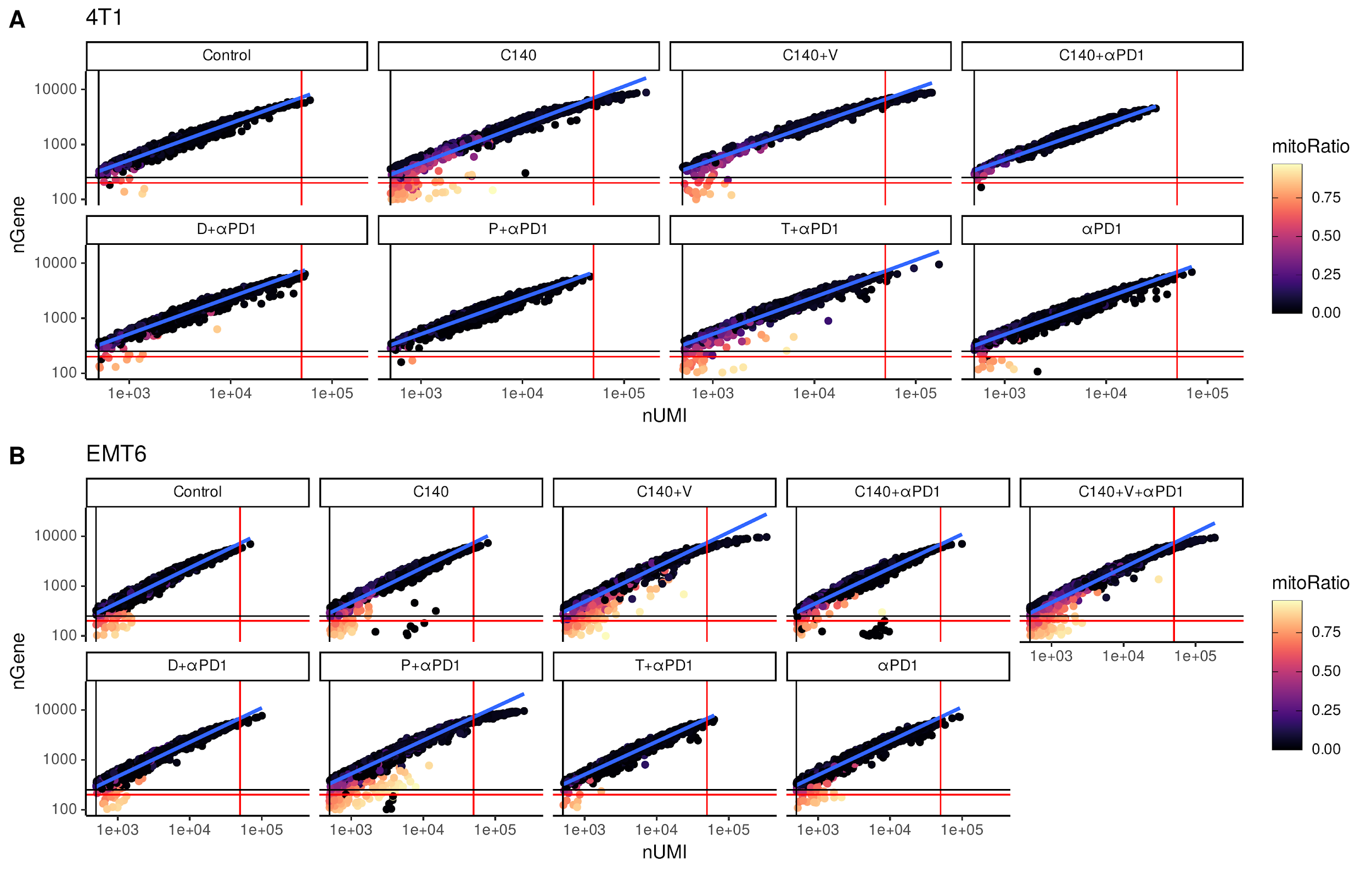


[**Fig.**](https://docs.google.com/document/d/1lLJdHMfHw569D3g2Zv8j2RN7NljR-NiECQhGmFpiZhI/edit#suppf_batche)**S**[**1**](https://docs.google.com/document/d/1lLJdHMfHw569D3g2Zv8j2RN7NljR-NiECQhGmFpiZhI/edit#suppf_batche)**. Single-cell RNA seq quality controls of two tumoral models.** Number of UMI plotted on x-axis versus number of genes on y-axis divided by condition for 4T1 (A) and EMT6 (B). Each dot represents a cell colour-coded by the mito ratio. Darker colours mean low mitochondrial expression, lighter colours indicate high mitochondrial genes. Vertical and horizontal red bars indicate the threshold used for number of genes (y-axis) and number of transcripts (x-axis).


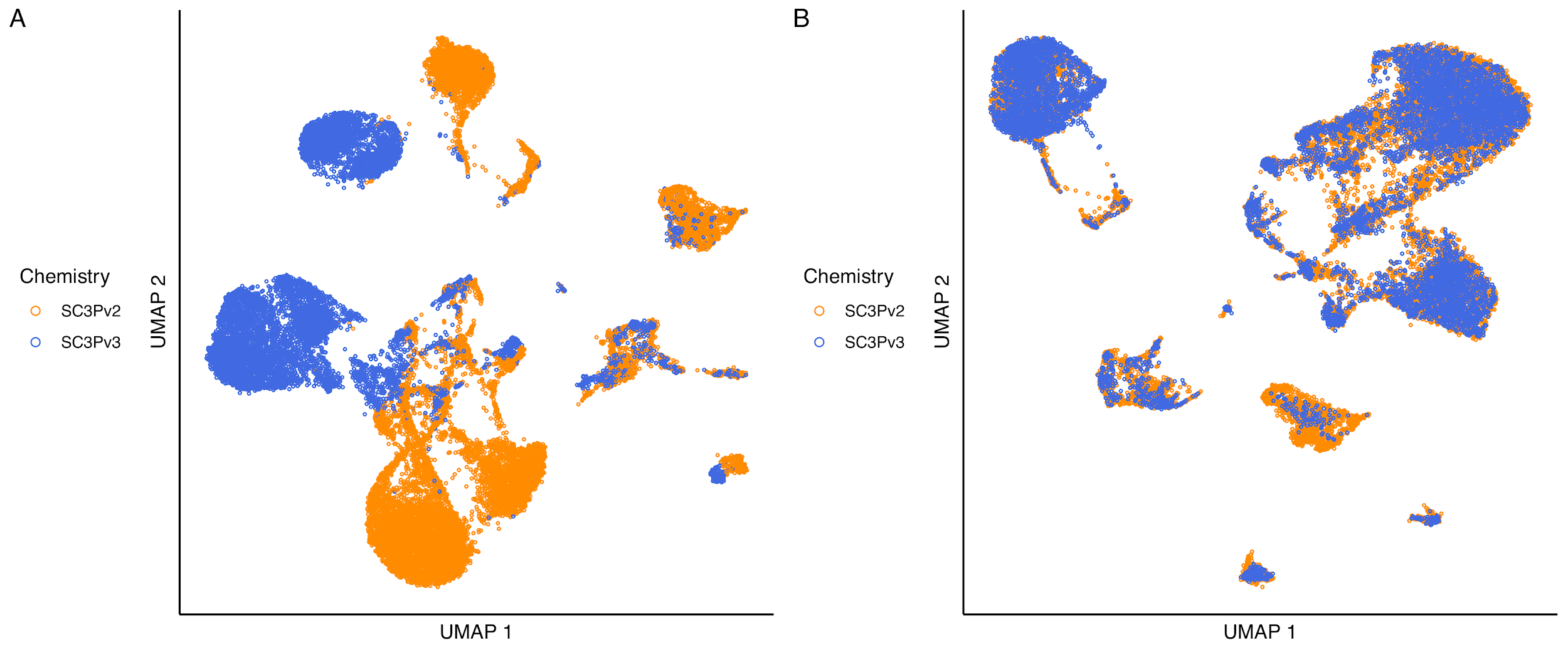


[**Fig. S2**](https://docs.google.com/document/d/1lLJdHMfHw569D3g2Zv8j2RN7NljR-NiECQhGmFpiZhI/edit#suppf_QC)**. Batch effect on 4T1 cell line.** A) UMAP showing batch effect on 4T1 cell line. Each cell is represented by a colored dot colored according to the 10X chemistry used. B) UMAP showing batch effect correction on 4T1. (Orange for V2 and blue for V3).


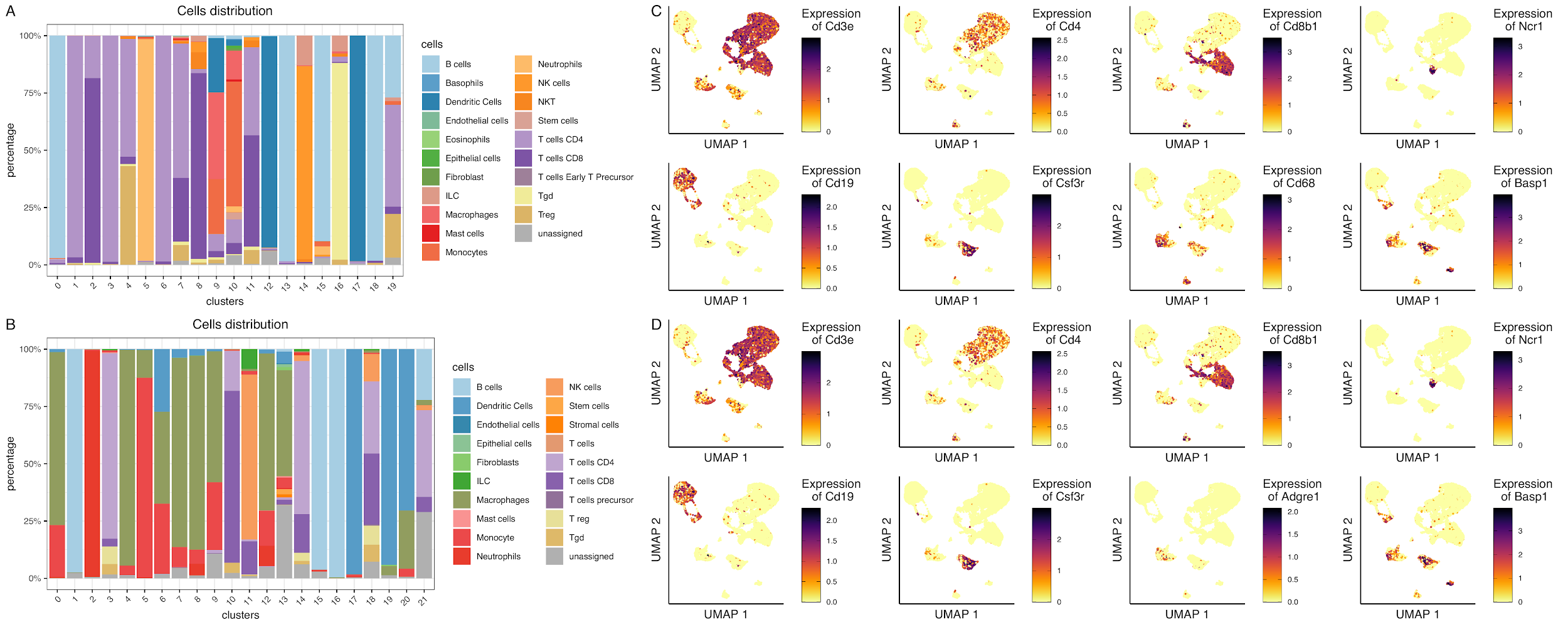


[**Fig. S3**](https://docs.google.com/document/d/1lLJdHMfHw569D3g2Zv8j2RN7NljR-NiECQhGmFpiZhI/edit#suppf_cellass)**. Cell Assignation.** Automatic cell populations assignment for 4T1 cell line (A) and EMT6 cell line (B). Clusters are arranged to the x-axis while the percentage of cells are signed on the y-axis. The colors are referring to the different types of cells automatically assigned by *SingleR*. C) UMAPs of known marker gene expression in 4T1 cell line. *Cd3e*, *Cd4*, *Cd8b1* genes were evaluated for T cells; *Ncr1* for NK cells; *Cd19* for B cells; *Csf3r* for neutrophils, *Cd68* for macrophages; *Basp1* for DC. Each dot represents a cell. The intensity of the colour (from yellow to dark violet) indicates the greatest expression of the specific marker gene. D) UMAP of known marker gene expression in EMT6 cell line. *Cd3e*, *Cd4*, *Cd8b1* genes were evaluated for T cells; *Ncr1* for NK cells; *Cd19* for B cells; *Csf3r* for neutrophils, *Adgre1* for macrophages; *Basp1* for DC. Each dot represents a cell. The intensity of the colour (from yellow to dark violet) indicates the greatest expression of the specific marker gene.


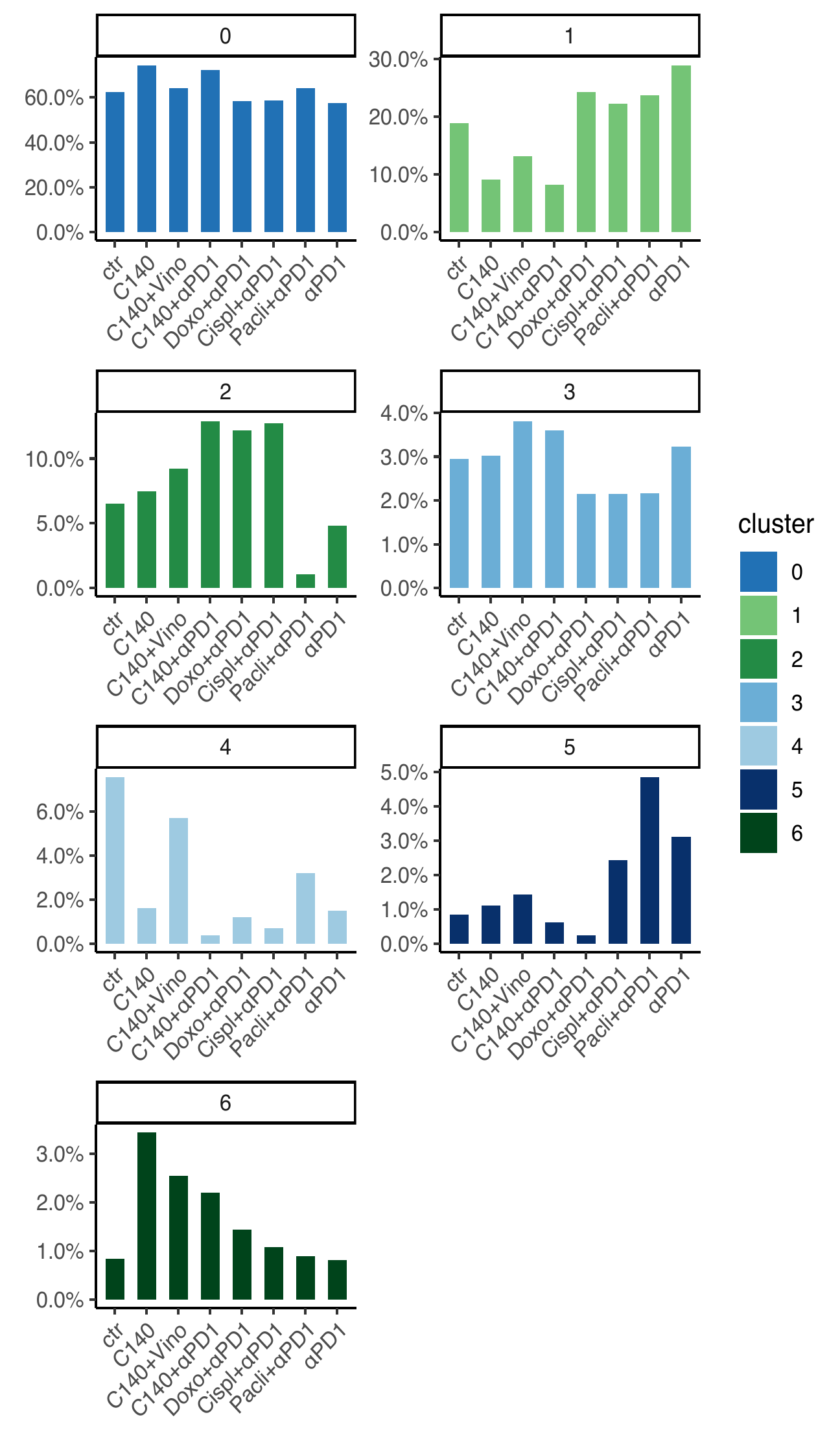


[**Fig. S4**](https://docs.google.com/document/d/1lLJdHMfHw569D3g2Zv8j2RN7NljR-NiECQhGmFpiZhI/edit#suppf_cd4)**. Cd4-like T cell sub-cluster variation.** Percentage of cells in each 4T1 Cd4-like T cell sub-cluster among different conditions. Bar graph shows on the x-axis the conditions, while the percentage of cells per cluster is plotted in the y-axis.


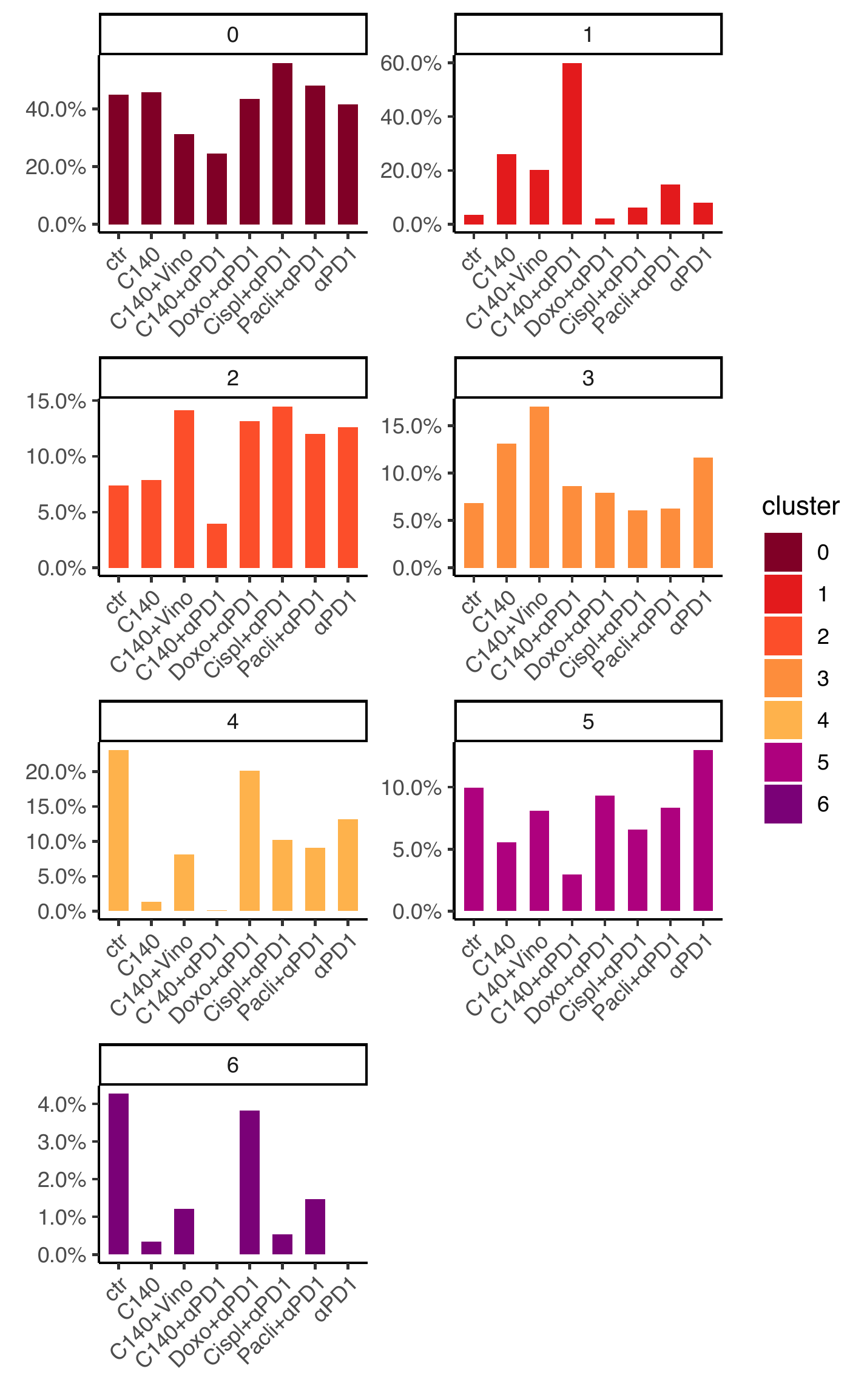


[**Fig. S5**](https://docs.google.com/document/d/1lLJdHMfHw569D3g2Zv8j2RN7NljR-NiECQhGmFpiZhI/edit#suppf_cd8)**. Cd8-like T cell sub-cluster variation.** Percentage of cells in each 4T1 Cd8-like T cell sub-cluster among different conditions. Bar graph shows on the x-axis the conditions, while the percentage of cells per cluster is plotted in the y-axis.


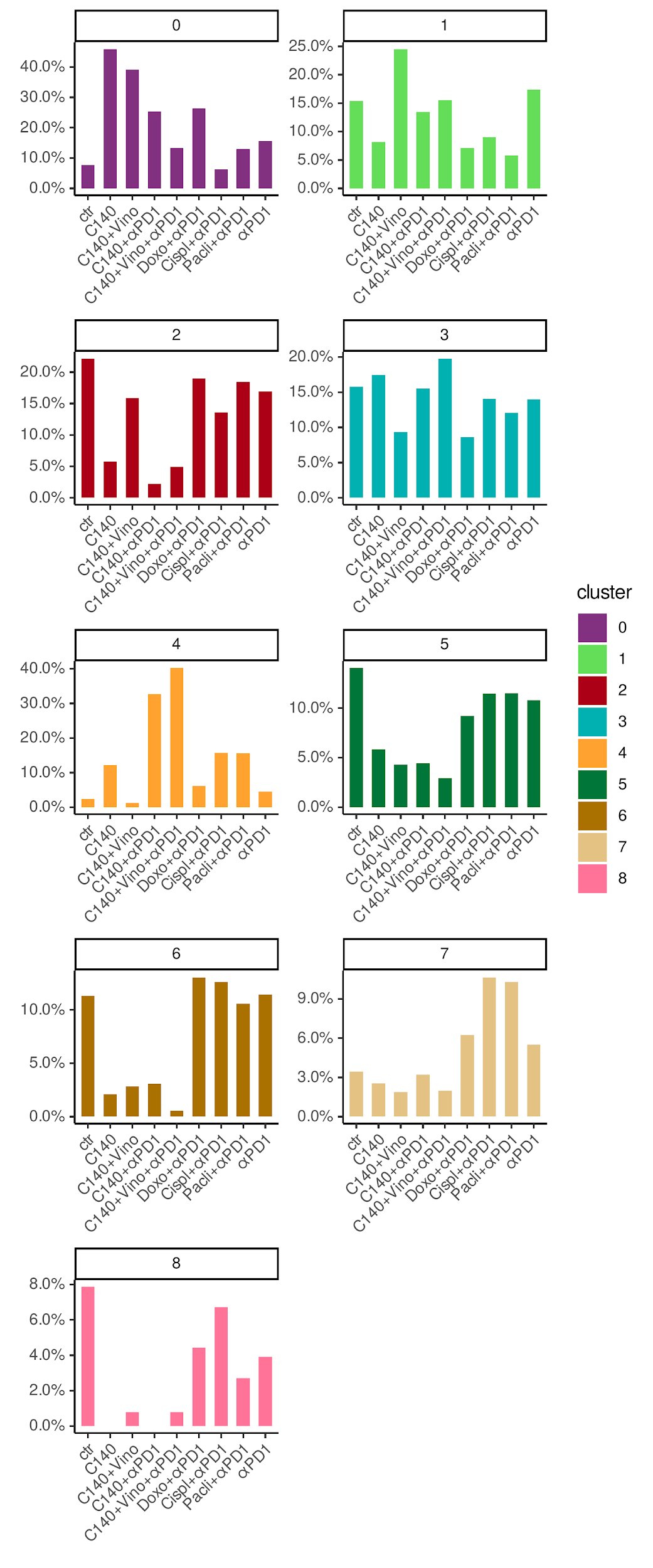


[**Fig. S6**](https://docs.google.com/document/d/1lLJdHMfHw569D3g2Zv8j2RN7NljR-NiECQhGmFpiZhI/edit#suppf_mac)**. Macrophage sub-cluster variation.** Percentage of cells in each EMT6 macrophages sub-cluster among different conditions. Bar graph shows on the x-axis the conditions, while the percentage of cells per cluster is plotted in the y-axis.


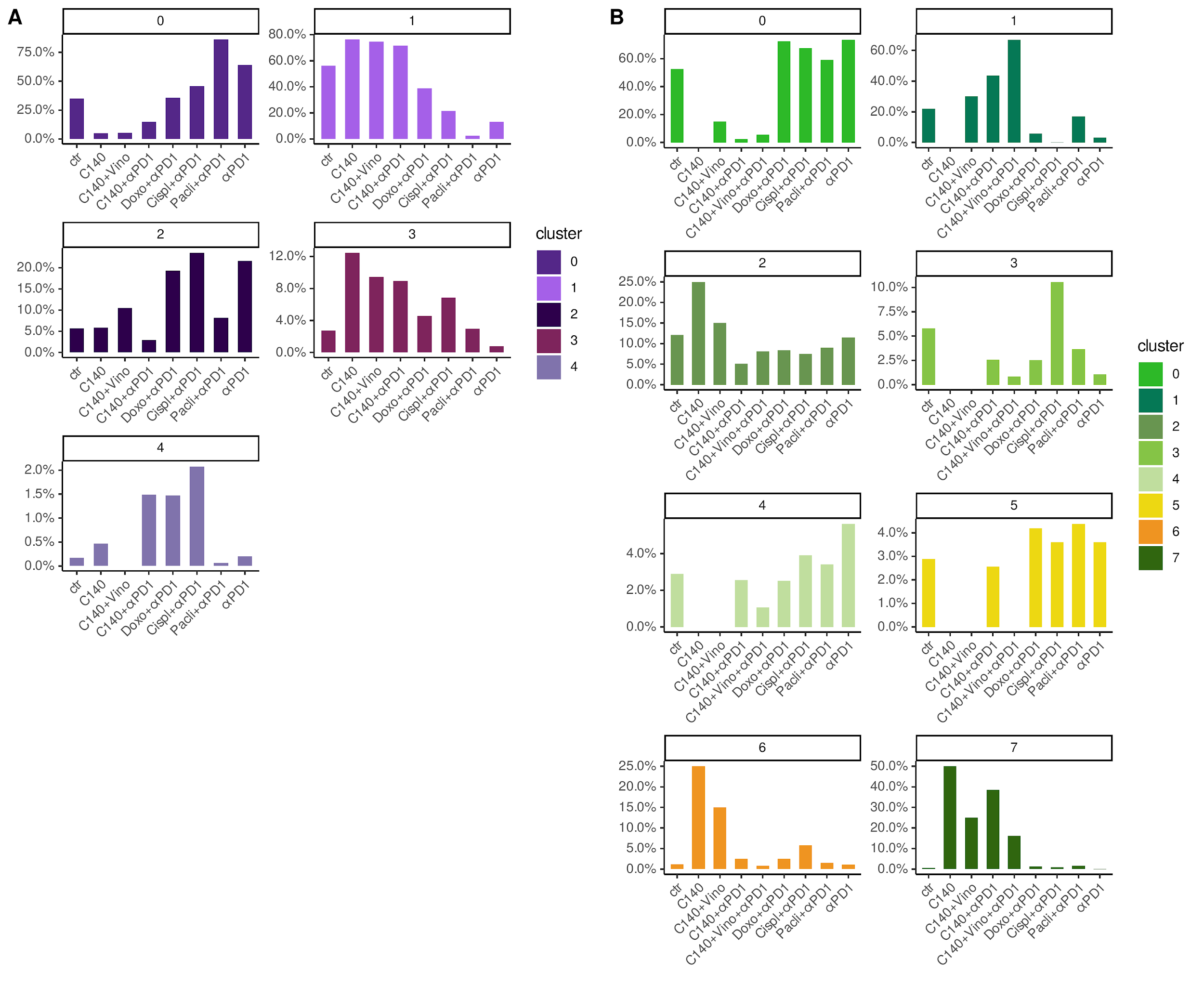


[**Fig. S7**](https://docs.google.com/document/d/1lLJdHMfHw569D3g2Zv8j2RN7NljR-NiECQhGmFpiZhI/edit#suppf_B)**. B cell sub-cluster variation**. Percentage of cells in B cell sub-clusters for 4T1 (A) and EMT6 (B) cell lines among different conditions. Bar graph shows on the x-axis the conditions, while the percentage of cells per cluster is plotted in the y-axis.

**Supplementary Table Legend (provided as Table excel)**

**Table S1**. Top 20 differentially expressed genes for the 4T1 cell line

**Table S2**. Top 20 differentially expressed genes for the EMT6 cell line

**Table S3**. Number of cells pre- and post- quality control for each condition in 4T1 cell line

**Table S4**. Number of cells pre- and post- quality control for each condition in EMT6 cell line
